# Impact of Grapevine Red Blotch Disease on Cabernet Sauvignon and Merlot Wine Composition and Sensory Attributes

**DOI:** 10.3390/molecules25143299

**Published:** 2020-07-21

**Authors:** Raul Cauduro Girardello, Monica L. Cooper, Larry A. Lerno, Charles Brenneman, Sean Eridon, Martina Sokolowsky, Hildegarde Heymann, Anita Oberholster

**Affiliations:** 1 Department of Viticulture and Enology, University of California, Davis, CA 95616-8749, USA; rgirardello@ucdavis.edu (R.C.G.); lalerno@ucdavis.edu (L.A.L.); cabrenneman@ucdavis.edu (C.B.); sseridon@ucdavis.edu (S.E.); hheymann@ucdavis.edu (H.H.); 2 Cooperative Extension, University of California, Napa, CA 94559-1311, USA; mlycooper@ucanr.edu; 3 Department of Life Science Technologies, Technische Hochschule Ostwestfalen-Lippe, 32657 Lemgo, Germany; martina.sokolowsky@th-owl.de

**Keywords:** grapevine red blotch disease, merlot, cabernet sauvignon, wine composition, phenolics, descriptive analysis

## Abstract

Grapevine red blotch disease (GRBD) is a recently identified viral disease that affects grapevines. GRBD has been shown to impact grapevine physiology and grape composition by altering specific ripening events. However, no studies have been reported on the impact of GRBD on wine composition and its sensory attributes. This study evaluated the impact of GRBD on wine primary and secondary metabolites, in addition to its sensory properties, when making wines from Cabernet Sauvignon and Merlot grapes during two seasons. Wines made with GRBD-impacted fruit were lower in ethanol content when compared to wines made with grapes from healthy grapevines. This was attributed to the lower total soluble sugar (TSS) levels of diseased grapes due to delayed ripening at harvest. GRBD impacted wine phenolic composition by decreasing anthocyanin concentrations and increasing flavonol concentrations in some instances. Additionally, proanthocyanidin concentrations were also consistently higher in GRBD wines compared to wines made from healthy fruit. Descriptive analysis demonstrated that GRBD can impact wine style by altering aroma, flavor, and mouthfeel attributes. However, the extent of GRBD impact on wine composition and sensory properties were site and season dependent.

## 1. Introduction

A recently recognized disease called grapevine red blotch disease (GRBD) is of great concern among grape growers, winemakers, and researchers [1]. Grapevine red blotch virus (GRBV), the causal agent of GRBD, was classified as a member of the *Geminiviridae* family, and its presence has been confirmed in the United States, Canada, Mexico, South Korea, and Switzerland [2,3,4,5,6]. More recently, it was found that the three-cornered alfalfa hopper (*Spissistilus festinus* Say) is a vector of GRBV under laboratory conditions [7].

Due to its recent discovery, only a small body of research regarding the impact of GRBD on grape and wine is available. The virus can impact grapevine foliar metabolism in Cabernet Franc and Cabernet Sauvignon [8] and affect ripening in Zinfandel grapes by suppressing specific ripening events, altering the expression patterns of transcription factors and causing hormonal imbalances [9,10]. Fruit composition is an important factor for wine quality, which is developed during the maturation of berries and tracked by viticulturists and winemakers. Traditional fruit maturation parameters are total soluble solids (TSS), mostly sugars measured as Brix, and organic acid concentration measured as titratable acidity (TA). Additionally, the grape phenolic composition is known to have an important impact on wine [11,12,13] and these parameters are affected by the presence of GRBV [14,15,16]. Sugar accumulation was reduced in GRBV-infected Cabernet Sauvignon, Cabernet Franc, and Merlot grapevines compared to healthy controls [1,17]. Berry sugar concentration influences wine style due to its conversion in ethanol during alcoholic fermentation. Previous research has shown that ethanol facilitates extraction of phenolic compounds, such as proanthocyanidin (PA) during alcoholic fermentation [18], and impacts the adsorption/desorption interactions between anthocyanins and solids present in wine during fermentation, such as grape cell walls, then affecting final anthocyanin concentration in wines [19]. In addition, ethanol has a direct impact on the sensorial perception of wine aroma, taste, and mouthfeel attributes [20].

Phenolic compounds present in the grape berry, such as flavan-3-ols, flavonols, hydroxycinnamic acids, anthocyanins, and PA, play important roles in fruit and wine quality. PA (also called condensed tannins) are oligomeric and polymeric flavan-3-ols present in grape seeds and skins and are of viticultural interest because they are the main component of astringency in red wines [21]. The second most abundant class of phenolics in red wines, anthocyanins, are responsible for the color in red grapes and wines. Anthocyanin concentration at harvest is an important factor and can influence grape prices and winemaking decisions [22]. In addition, hydroxycinnamic acids are usually the most abundant class of phenolics in free-run juice and, consequently, in white wines [12]. Flavonols are less abundant, but they play an essential role in the color stability of wines through co-pigmentation with anthocyanins [23].

Studies demonstrated that phenolic compounds present in grape berries may be affected by grapevine viral diseases, thus leading to changes in wine’s sensory attributes. For example, Merlot wines made from fruit infected with grapevine leafroll disease (GLD) presented significant decreases in anthocyanin and PA concentrations [24]. Descriptive analysis (DA) on wines made with grapes from GLD-infected and healthy grapevines demonstrated a significant impact on the perceived color, aroma, and astringency of the wines due to disease status [24]. However, the impacts of GRBD on wine composition and sensory properties are still unknown.

In this study, the impacts of GRBD on wine composition and sensory properties were investigated in two grapevine cultivars (Cabernet Sauvignon and Merlot) across three vineyards over two seasons. The goal was to determine potential trends due to GRBD on wine composition across site and season, and this is the follow-up study of a recently published paper regarding the impacts of GRBD on grape composition [16].

## 2. Results

The current study is a screening of different vineyards to determine the potential impact of GRBD on wine composition and sensory attributes. It complements a prior study that focused on the impact of GRBD on grape composition, utilizing the same grapes used in this study, in addition to other vineyards that could not be used for winemaking due to the limited amount of grapevines available [16]. Additionally, due to vine removal (rouging), wines were made from three different vineyards in 2014 and only one in 2015. This is the first study comparing the composition and sensory characteristics of wines made with fruit from asymptomatic “RB (−)” and symptomatic “RB (+)” grapevinesfor Grapevine Red Blotch Disease (GRBD) from each vineyard site, and it will enable us to determine whether there are any consistent impacts, regardless of site differences.

### 2.1. Must Composition

Basic chemical juice composition of grapes harvested from RB (−) and RB (+) grapevines is presented in Table 1.

The main impact of GRBD was observed in the juice sugar content. At all sites and in both study years, RB (+) juice was lower in TSS (expressed as Brix) when compared to RB (−). Consistently, RB (+) juice also had lower pH and higher TA when compared to RB (−) grapes. The differences observed in juice TSS impacted the fermentation profile for each RB (−) and RB (+) wine, which is presented in the supplemental data section (Supplemental Figure S1). All the fermentations progressed similarly, regardless of whether the wine was made from RB (−) or RB (+) grapes, although RB (+) wines reached 0 Brix one day earlier than RB (−) wines, except for Cabernet Sauvignon—Site 2 (CS2) wines in 2014. These differences were expected since RB (−) musts had higher TSS than RB (+) at the onset of fermentation.

### 2.2. Wine Composition

Table 2 presents the basic chemical composition of RB (−) and RB (+) wines made in 2014 and 2015. Due to the lower sugar concentrations in the RB (+) musts, RB (+) wines had lower ethanol percentages when compared to RB (−) wines, regardless of site, cultivar, or season. On the other hand, GRBD did not have a consistent effect on wine’s pH. However, TA was adjusted during winemaking to six g/L with tartaric acid addition, if needed. In the must, CS2 RB (−) grapes had slightly higher pH at harvest when compared to RB (+) (Table 2), following a similar trend in the final wines.

### 2.3. Impact on Phenolic Composition

**Figure 1:**
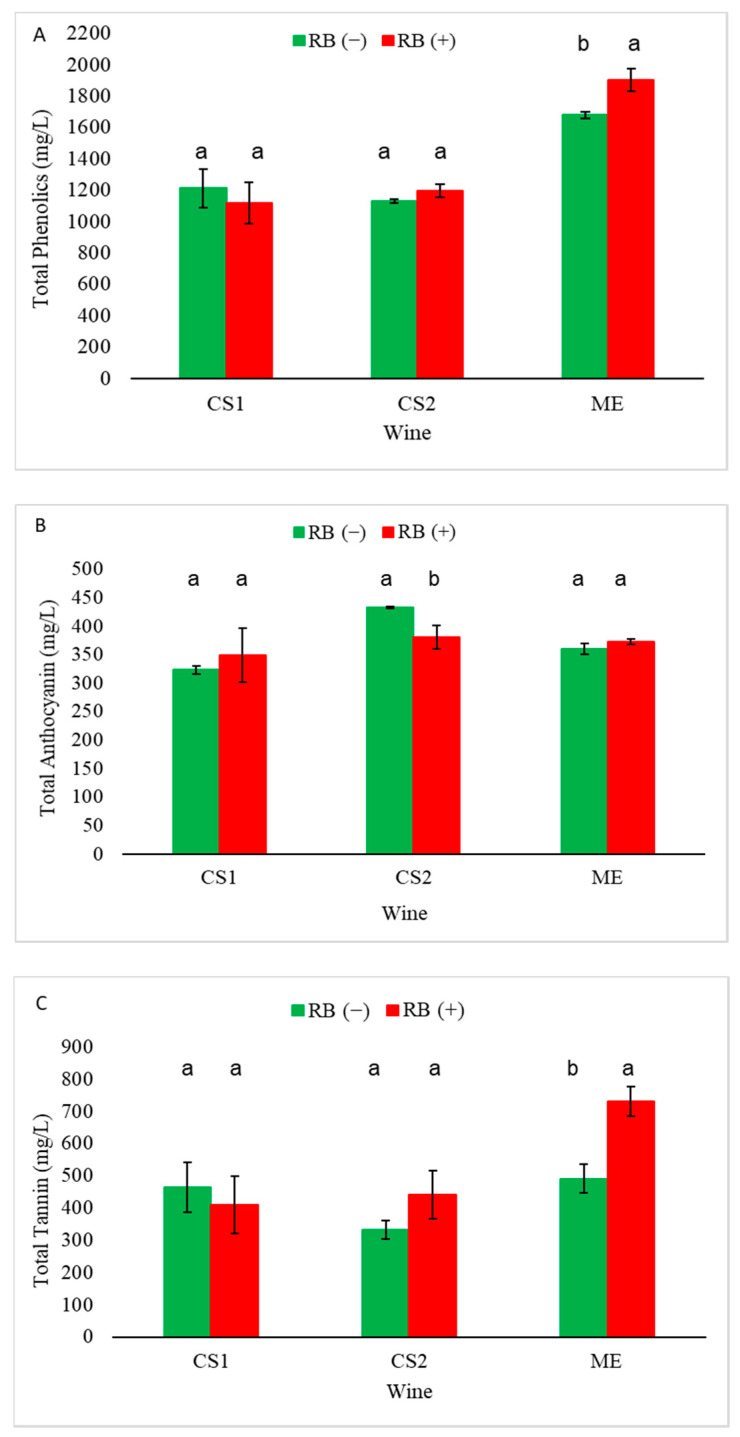
Cabernet Sauvignon Site 1 (CS1), Cabernet Sauvignon Site 2 (CS2), and Merlot (ME) total phenolics (**A**), total anthocyanins (**B**), and total tannin (**C**) concentration of 2014 wines (n = 3 for S2 and n = 2 for CS1 and ME, *p* < 0.05). Statistical differences are expressed as letters and indicate significant differences in the LSD test for each site.

Wine phenolic analysis demonstrated that GRBD impacted the concentration of different classes of phenolics. Figure 1 shows the impact of GRBD on total phenolics, total anthocyanins, and total tannins. Concerning wines made in 2014, Merlot (ME) RB (+) wines had a higher concentration of total phenolic and total tannins when compared to ME RB (−). On the other hand, both Cabernet Sauvignon wines (Cabernet Sauvignon—Site 1 (CS1) and CS2) did not show any statistical difference regarding total phenolic and total tannin concentrations between RB (−) and RB (+). However, GRBD impacted total anthocyanins significantly for site CS2 by decreasing its concentration in RB (+) wines.

Thus, for total phenolics, anthocyanins, and tannins, CS1 RB (+) wines did not show any statistical differences when compared to CS1 RB (−) wines. Similar trends were found in 2015 when compared to the 2014 season for total phenolic, total anthocyanin, and total tannin concentrations for site CS2 wines (Figure S2). One difference was that the tannin concentration in CS2 RB (+) wines was significantly higher than CS RB (−) wines (Figure S2).

Monomeric phenolic profiles were determined in wines made from RB (−) and RB (+) grapes by reversed phase-high performance liquid chromatography (RP-HPLC). Monomeric anthocyanin concentrations of RB (−) and RB (+) wines produced in 2014 and 2015 are presented in Table 3. For CS2 wines for both seasons, the concentration of most of the monomeric anthocyanin forms were significantly lower in RB (+) wines when compared to RB (−), including the two most abundant forms, malvidin-3-glucoside and malvidin-3-acetylglucoside. Consequently, total anthocyanin concentrations were lower in RB (+) wines compared to RB (−) wines. A similar trend was observed for ME wines, in which most of the monomeric anthocyanin forms were lower in ME RB (+) wines in comparison to RB (−). However, the three malvidin-3-glucoside forms were lower in ME RB (−) wines when compared to ME RB (+) wines, resulting in total anthocyanin concentrations which were not significantly different. Polymeric pigments were also impacted by GRBD (Table 3). RB (+) wines from both Cabernet Sauvignon sites (CS1 and CS2 for both seasons) were significantly lower in polymeric pigment concentration when compared to RB (−) wines. The same was not true for ME wines, in which no significant differences were found between RB (−) and RB (+) wines.

Concerning other classes of phenolics analyzed, the impact of GRBD on wines made with RB (+) grapes affected all three sites and varied depending on the class of phenolics analyzed. The impact on flavan-3-ol compounds was dependent on the cultivar and site. Wine from the same cultivar (Cabernet Sauvignon) but grown at a different site and under different conditions (such as rootstock, soil, and age) were impacted differently. There was no difference in catechin, epicatechin, and total flavan-3-ol concentrations between CS1 RB (−) and CS1 RB (+) wines. On the other hand, CS2 RB (+) wines contained significantly lower concentrations of catechin and total flaval-3-ols, when compared to CS2 RB (−) wines in 2014. In contrast, ME RB (+) wines contained higher concentrations of catechin and total flavan-3-ols compared to ME RB (−) wines.

Differently to the results observed for flavan-3-ols, the impact of GRBD on flavonol concentration exhibited more consistent trends. For two of the sites (CS2 and ME) analyzed in 2014, wines from RB (+) grapes had a higher concentration of flavonols when compared to RB (−). For CS2, concentrations of quercetin-3-galactoside, quercetin-3-glucoside, and quercetin were significantly higher in RB (+) wines compared to RB (−) wines. A similar trend was observed in ME wines, where the concentration of quercetin-3-glucoside was higher in wines made from RB (+) grapes than the wines made from RB (−) grapes.

### 2.4. Proanthocyanidin Composition of Wines

The differences in PA composition and concentration between RB (−) and RB (+) wines for each site were determined within one month of sensory evaluation (five months after bottling) by phloroglucinolysis (Table 4).

Numerous significant differences were found between RB (−) and RB (+) wines in both seasons. RB (+) wines had significantly higher PA concentration when compared to RB (−) wines, regardless of the cultivar, site, or season.

The mean degree of polymerization (mDP) and average molecular weight (MW) were impacted only in wines from one vineyard. For CS2, PA from RB (+) wines had higher mDP’s when compared to RB (−) wines in 2014. However, CS2 wines from 2015 showed no significant differences in mDPs. Regarding the degree of galloylation (Galloylation%), which expresses the percentage of monomers derivatized with gallic acid, ME RB (−) wines had higher degrees of galloylation when compared to ME RB (+).

### 2.5. Untargeted Metabolomics Analysis

Table 5 and Table 6 show the identified metabolites measured by metabolomics analysis in wines from the 2014 and 2015 seasons, respectively. For each site, metabolite levels that were significantly higher in RB (+) wines when compared to RB (−) wines are highlighted in light grey, and compounds that were significantly higher in RB (−) wines when compared to RB (+) wines are highlighted in dark grey. The levels of ninety-seven out of 158 (61%) and 50 out of 157 (31%) known compounds analyzed in 2014 and 2015, respectively, were significantly different between RB (−) and RB (+) wines for at least one site.

Most of the changes were observed in CS1 RB (+) wines, which had significantly decreased levels of 36 metabolites in comparison to CS1 RB (−) wines, and CS2, which had the levels of 47 metabolites significantly reduced and the levels of 27 metabolites significantly increased in RB (+) wines when compared to RB (−) (2014 season). However, the impacts on ME wines showed a different trend, especially regarding carboxylic acids, in which RB (+) wines had higher levels of 14 metabolites when compared to RB (−) wines, but minimal impact on polyols, lipids, and oligosaccharides (Table 5). ME RB (−) and RB (+) grape composition differences were minimal with respect to TSS and pH, but the highest among the sites in 2014 regarding TA and malic acid (higher level in RB (+) grapes), which may be related to the increased levels of carboxylic acids in RB (+) wines. Regarding CS2 wines in 2015, the only site where the study could be replicated in two seasons, RB (+) wines had lower levels of 38 metabolites when compared to RB (−) wines (Table 6).

Trends regarding the impact of GRBD on monosaccharide and oligosaccharide levels were also observed in the wines. In general, RB (−) wines had higher levels of some monosaccharides and oligosaccharides compared to RB (+) wines, especially for site CS1 (in 2014) and site CS2 (in 2014 and 2015). For example, trehalose levels were demonstrated to be higher in RB (−) than RB (+) wines (except for site ME).

Although metabolomics analysis demonstrated that in general RB (−) wines had higher levels of monosaccharides than RB (+), these observations were more dependent on the compound analyzed and site. For example, the levels of only seven monosaccharides were statistically different between ME RB (−) and ME RB (+) wines in 2014 (three higher and four lower in RB (−) wines compared to RB (+) wines). On the other hand, CS2 RB (−) wines had mostly higher levels of monosaccharides than CS2 RB (+) wines in both seasons investigated (Table 5 and Table 6).

No clear trends were observed for carboxylic acids in 2014 (Table 5). Of the 36 compounds identified in ME wines, RB (+) wines had higher levels of 14 carboxylic acid compounds in comparison to RB (−) wines. On the other hand, CS1 RB (−) wines had higher levels of carboxylic acid compounds when compared to CS1 RB (+) wines. Site CS2 demonstrated a different trend, with almost half of the carboxylic acid metabolites having significantly higher values in RB (−) wines (13 compounds), while the other half were higher in RB (+) wines (13 compounds). A different trend was observed in 2015 for the same site (CS2) (Table 6), with 10 carboxylic acids with significantly higher values in CS2 RB (−) wines and only two higher in CS2 RB (+) wines.

No clear trends were found concerning differences in disease status of the grapes, regarding wine polyol and lipid contents. For polyols, the most impacted wines were CS2, which showed a heterogeneous impact in 2014, with significantly higher levels of five compounds, and lower levels of three compounds in RB (+) wines in comparison to RB (−) wines (Table 5). This disagrees with the results found in CS2 wines in 2015, which mostly showed higher levels of five polyol metabolites in RB (−) wines (Table 6). These results confirm the observations in most of the chemical and phenolic data, which shows that the impacts of GRBD depended on the site and season.

### 2.6. Impact of GRBD on Wine Sensory Properties

Descriptive analysis (DA) demonstrated that GRBD impacted not only the chemical composition but also the sensory properties of wines made with fruit from GRBD affected vines. A list of 26 attributes in 2014 and 26 attributes in 2015 (aroma, taste, and mouthfeel) was generated by panel members to describe the wines. Supplemental Table S2 presents the mean score of each attribute and statistical differences between RB (−) and RB (+) wines for each site studied in 2014 and 2015.

**Figure 2:**
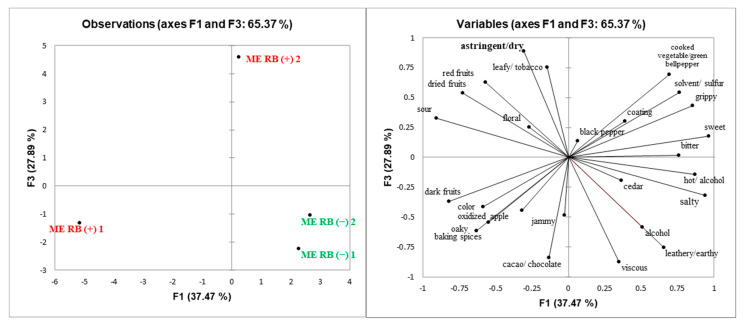
Score (**left**) and loadings (**right**) plots of principal component analysis (PCA) of ME RB (−) and RB (+) 2014 wines. Attributes found to be significantly different by ANOVA (*p* < 0.05) are in bold.

**Figure 3:**
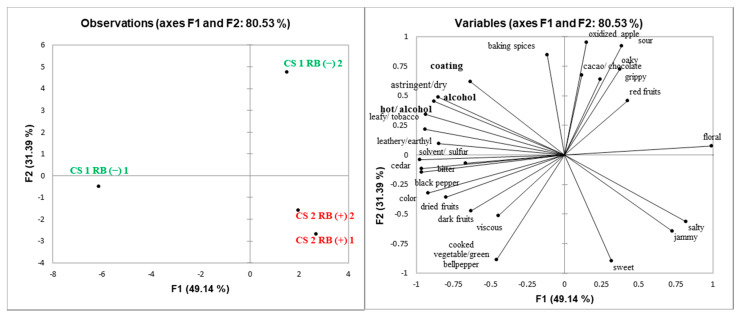
Score (**left**) and loadings (**right**) plots of principal component analysis (PCA) of CS1 RB (−) and RB (+) 2014 wines. Attributes found to be significantly different by ANOVA (*p* < 0.05) are in bold.

**Figure 4:**
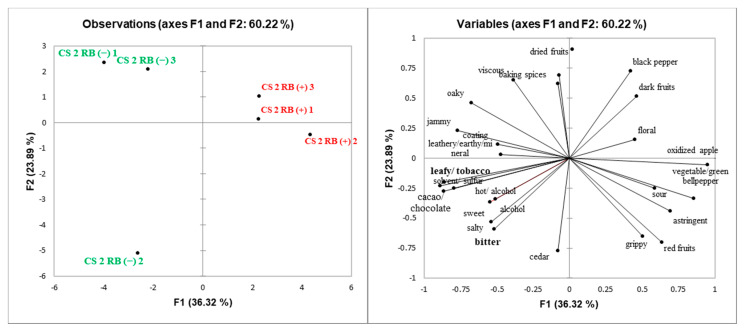
Score (**left**) and loadings (**right**) plots of principal component analysis (PCA) of CS2 RB (−) and RB (+) 2014 wines. Attributes found to be significantly different by ANOVA (*p* < 0.05) are in bold.

**Figure 5:**
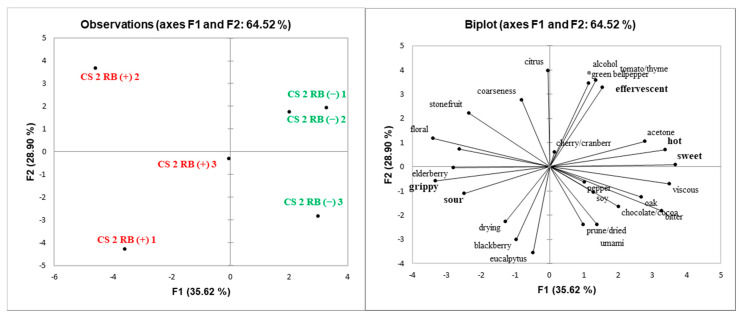
Score (**left**) and loadings (**right**) plots of principal component analysis (PCA) of CS2 RB (−) and RB (+) 2015 wines. Attributes found to be significantly different by ANOVA (*p* < 0.05) are in bold.

Principal component analysis (PCA) was performed to explore potential relationships among sensory attributes and GRBD status of wines (Figure 2, Figure 3, Figure 4 and Figure 5). Spatial separation between RB (−) and RB (+) wines were observed for all the wines. Wine composition indicates that GRBD delays grape ripening, resulting in RB (−) wines with higher ethanol content, which had a significant impact on wines’ sensory properties. In general, for all three sites studied in 2014 and the one site in 2015, it was demonstrated that RB (−) wines highly correlated with sensory attributes, such as “alcohol” aroma and “hot” mouthfeel. These attributes are directly related to ethanol concentration, agreeing with results found by King et al. (2013). On the other hand, “sour” was more related to RB (+) wines for ME (2014) and CS2 (both 2014 and 2015) wines, which agrees with higher TA and lower pH in the wines due to delayed grape ripening and also agrees with the lower ethanol concentration of RB (+) wines, which likely enhanced sourness perception [25].

In general, the mouthfeel attribute “astringency” was positively correlated with RB (+) wines, except for site CS1. Both methodologies used to determine tannin concentration (protein precipitation and phloroglucinolysis) demonstrated that CS2 RB (+) (in both 2014 and 2015 seasons) and ME RB (+) wines had higher tannin concentration than RB (−) wines. In addition, high ethanol concentration can suppress astringency [26], another factor that may have influenced the perception of “astringency” in RB (+) wines (Figure 2, Figure 4 and Figure 5). The exceptions were wines from site CS1, where RB (+) wines rated lower in astringency compared to RB (−) wines although it contained more PA. This may potentially be due to the higher percentage of tannin galloylation in the RB (−) wines contributing to drying, which correlates strongly with astringency [9].

## 3. Discussion

Grapes from diseased grapevines had lower sugar content when compared to healthy grapes as a result of delayed grape ripening due to GRBV infection as demonstrated in previous studies [10,15,16] which resulted in RB (+) wines with lower ethanol content when compared to RB (−) wines. However, the extent of the disease’s impact on sugar accumulation was site and seasonal dependent. A larger difference was observed between RB (−) and RB (+) wines for CS1 in comparison to CS2 and ME sites in the 2014 season. In 2015, the difference between CS2 RB (−) and CS2 RB (+) wines was more significant when compared to the previous year (2014), which suggests that the impact of GRBD is variable depending on the season and thus environmental influences. The 2015 season was warmer than 2014 (Supplemental Table S1) indicating that temperature may influence the impact of GRBD on grapevines. Previous work has demonstrated that plant defense mechanisms, such as short-interfering RNAs (siRNAs), against geminiviruses infection are influenced by temperature and can significantly reduce symptom severity at 25 °C when compared to 30 °C in cassava (*Manihot esculenta*, Crantz) and tobacco (*Nicotiana benthamiana)* [27]. Therefore, we suggest that due to the warmer 2015 season, the impact of GRBD was greater than in 2014, resulting in larger differences between RB (−) and RB (+) wines in 2015.

The higher pH in CS2 RB (−) wines for both years studied may be partially explained by the potassium (K) content, which is known to be positively correlated to pH in wines [28]. Unfortunately, K concentrations were not measured in RB (−) and RB (+) wines. However, an overall grape sample taken from CS2 vineyard at harvest in 2015 demonstrated that RB (−) and RB (+) grapes had 2110 mg/L and 1970 mg/L of K, respectively. With respect to wine acidity, even though TA was adjusted prior to fermentation, CS2 RB (−) and CS2 RB (+) wines were significantly different in 2014 and 2015, which also may have contributed to the higher pH values in CS2 RB (−) wines when compared to CS2 RB (+) wines (Table 2). However, this difference was small enough that it will not impact the wines’ sensory perception [29].

Differences in phenolic composition between RB (−) and RB (+) wines varied depending on the site and season. In 2014, the impact of GRBD depended on the site with only CS2 showing a significant decrease in anthocyanin concentration in RB (+) wines compared to RB (−) wines. Additionally, CS1 and CS2 exhibited significant decreases in polymeric phenols (tannin) due to RB disease status whereas ME was not impacted for that variable. GRBD did decrease anthocyanin concentration in the CS2 wines in both seasons studied. However, catechin and total flavan-3-ols were only significantly decreased in CS2 RB (+) wines in one season.

On the other hand, polymeric pigment concentrations were shown to be decreased in most of RB (+) wines (except for ME RB (+) wines (Table 3). It is known that polymeric pigments are important for wine aging due to its role in color stability, which can account for 50% of color density within the first year of aging [30]. Therefore, results found in this study suggest that wines made from RB (+) grapes may have lower color intensity over time than those made from RB (−) grapes due to their decreased anthocyanin and polymeric pigment concentrations.

The higher concentration of flavonols in CS2 RB (+) wines in 2014 compared to RB (−) wines could be due to either alteration in grape berry biochemistry due to GRBV infection or due to the reduced vigor of diseased grapevines, which increased sun exposure on RB (+) grape berries. It has been demonstrated that viral diseases can reduce grapevine vigor and its normal growth [31] and studies have shown that the biosynthesis of flavonols in grape berries is highly dependent on sun exposure [12]. In addition, it was found that GRBD decreased pruning weight in CS and shoot length in ME vines [1,17]. Therefore, it is possible that a decrease in leaf area and canopy volume, resulting in higher sun exposure of clusters from RB (+) grapes could partially explain these results. It has been demonstrated that CS grapes infected with GLRaV-3 had a higher content of quercetin (one of the most abundant flavonols in grapes) when compared to grapes from non-infected vines [32].

It is important to note that the extractability of phenolic compounds from grapes into wine is not easy to predict. Many factors influence the extraction of phenolics from grape skins and seeds during winemaking, such as the degree of maturity in which the grapes were harvested, maceration conditions, and ethanol produced during fermentation [11,33,34,35]. After extraction, phenolic compounds may adsorb to yeast and grape berry cell walls, which can impact their final concentration in the wine [36]. *Vitis vinifera* cultivars vary regarding their cell wall composition and morphology, and, consequently, the extractability of phenolic compounds may be different [37,38]. At present, the impact of GRBD on the composition and morphology of skins cell wall, where most of the phenolic compounds of winemaking interest are located, is not known.

One of the most important factors responsible for PA extraction during winemaking is ethanol concentration. It has been found that higher ethanol production in wines during fermentation increases the extraction of skin and seed PA significantly [18]. However, RB (+) wines consistently contained lower ethanol concentrations when compared to RB (−) wines due to delayed ripening in RB (+) grapes. All other winemaking parameters that could impact phenolic extractability, such as maceration and pump-over regime, were standardized among different treatments. Therefore, the higher PA concentration in RB (+) wines compared to RB (−) wines was related to PA content in the grape berries due to GRBV infection. Plants have developed defense mechanisms under abiotic and biotic stresses, such as viral infections. Flavonoid biosynthesis is often enhanced under the influence of several types of stress, such as pathogens [39]. Additionally, it has been shown that Merlot grapevines infected by GLRaV-3 had higher PA content in leaves when compared to leaves from healthy vines [40]. The impacts of GRBD on grape composition were studied on the grapes used to produce the wines analyzed in the current study [16]. It was found that skins from RB (+) berries had a higher concentration of PA when compared to RB (−) berries. It is well-documented that PA extractability is higher from skins than seeds during red wine fermentation [41], especially when no extended maceration is employed. Therefore, the higher concentration of PA in RB (+) compared to RB (−) wines is likely due to increased synthesis of PA in grape skins in response to stress caused by GRBV infection. Tannins are known to be part of the defense mechanism in plants [39].

Studies performed on *Vitis vinifera* L. cv. Zinfandel grapes during one season showed that GRBV was able to inhibit ripening-associated pathways, such as a reduced metabolic flux in the central and peripheral phenylpropanoid pathways, impacting the expression of transcription factors that are part of the biosynthesis of anthocyanins [9]. However, our results demonstrated that the impact of GRBD on wine phenolic composition is variable and depended on site and seasonal factors. Similarly to GRBD, previous studies on grapevine leafroll disease showed a seasonal impact on the effect of the disease on both basic chemical and phenolic composition of grapes and wines [24]. In addition, it is known that mineral nutrition, yield, vigor, stage of development, environmental conditions, and biotic and abiotic factors have a strong impact on grape phenolic accumulation and composition [42].

As demonstrated in this study, RB (−) grapes consistently had higher sugar content than RB (+) grapes. It has been shown that increasing must sugar concentration as a result of advanced grape maturity resulted in significant effects on yeast metabolism during fermentation. As the production of ethanol by yeast increased, there were concomitant increases in most yeast-derived metabolites [43]. Amino acid levels were shown to be strongly impacted by GRBD. Amino acids present in wine originate from different sources: those indigenous to the grape that can be partially or entirely metabolized by yeasts during fermentation; and those excreted by live yeasts at the end of fermentation or released by proteolysis during the autolysis of dead yeasts [44]. In both cases in which ethanol content differences were the largest between RB (−) and RB (+) wines (CS1, 2014 and CS2, 2015), RB (−) wines had higher levels of specific amino acids when compared to RB (+) wines (Table 5 and Table 6). Yeast activity and reproduction were likely extended due to the higher sugar content at the onset of fermentation and longer fermentation (as shown in supplemental data—Figure S1) in RB (−) wines, thus resulting in higher biosynthesis and later release of amino acids during yeast autolysis. Amino acids, such as asparagine, beta-alanine, alanine, glutamine, threonine, glutamine, valine, and glycine, were shown to be present at a higher level in at least one of the RB (−) wines when compared to RB (+) wines. The same amino acids were shown to be released during yeast autolysis in model wine [45].

In general, RB (−) wines contained higher levels of monosaccharides and oligosaccharides compared to RB (+) wines. For instance, trehalose is known for its role as a reserve carbohydrate in yeast, but it is also associated with the protection of cells against many environmental stressors, including ethanol stress [46]. Therefore, it is possible that RB (−) wines contained higher levels of this compound after yeast autolysis due to the larger biomass of yeast produced during fermentation (due to higher sugar contents at the onset of fermentation in RB (−) grapes) and also due to the higher ethanol content (Table 6), which potentially induced trehalose biosynthesis for yeast protection against ethanol. Another example is that RB (−) wines also exhibited higher levels of cellobiose compared to RB (+) wines at all the sites. Cellobiose, a disaccharide of glucose, is the repeating unit of cellulose, which is known to be part of grape berry cell walls [47]. Previous studies have demonstrated that GRBD delays grape ripening [1,10,15,16], which potentially alters cell wall modifications in grapes. During ripening, a substantial weakening of primary cell walls and degradative changes to cell wall polysaccharides occur. These changes in cell wall architecture, combined with the increased pore size, make the cell wall a much more open structure, increasing the accessibility of enzymes responsible for cell wall degradation at later ripening stages, and decreasing limitation to cell wall disassembly [48]. Therefore, it is possible that RB (−) wines had higher levels of cellobiose than RB (+) wines due to the advanced stage of ripening of RB (−) grapes, which may have facilitated cellulose hydrolysis during fermentation.

The delayed grape ripening caused by GRBD could also potentially impact polysaccharide content in wines. It has been shown that wines made with grapes harvested at seven weeks after veraison had higher amounts of polysaccharides than wines made with grapes harvested three weeks after veraison [49]. One hypothesis is that because GRBD delays grape ripening, lower levels of polysaccharides are being released into the wine during fermentation/maceration. Since part of the monosaccharides and oligosaccharides in wines originate from hydrolyzed polysaccharides during fermentation and aging, RB (+) wines ended up, in general, with lower levels of monosaccharides and oligosaccharides than RB (−) wines.

No clear GRBD impact was observed for carboxylic acids in wine, with vineyard site and season having a larger influence. Many carboxylic acids present in wines, such as citric acid, succinic acid, malic acid, and pyruvic acid, are formed as products of alcoholic fermentation during the glycolysis process through the Krebs cycle, which uses hexose sugars as substrate [13]. Thus, musts with fewer hexose sugars (mainly glucose, fructose, and galactose in grapes) at the start of fermentation could result in wines with less carboxylic acids. A trend of increased carboxylic acid levels was observed for wines made from grapes which had the largest differences between RB (−) and RB (+) must TSS and thus wine ethanol content (CS1, 2014 and CS2, 2015).

The sensorial differences between RB (−) and RB (+) wines demonstrated by DA strongly correlated with the chemical analysis of the wines. The chemical composition of CS1 wines indicates that the difference in ethanol concentration between RB (−) and RB (+) (14.6% and 13.0%, respectively—Table 2) was large enough to be perceived by panelist regarding “alcohol” aroma and “hot” mouthfeel. In a study with Riesling wines, judges rated the attribute “heat” higher in wines with 13.6% (*v*/*v*) ethanol than in wines with 12.6% (*v*/*v*), demonstrating that a difference of 1% (*v*/*v*) in ethanol content in wines is large enough to be perceived by a trained consumer panel [50]. Ethanol also has been shown to play a synergistic role with catechin, which enhanced differences between RB (−) and RB (+) wines regarding “bitter” taste. Chemical and phenolic analysis showed that CS2 RB (−) wines had higher ethanol content and catechin concentration when compared to CS2 RB (+) wines. Studies found that a higher concentration of catechin and ethanol enhances bitterness in wine [20,25].

GRBD had the smallest impact on ME as seen by chemical analysis of the wines (Table 4, Table 5, Table 6 and Table 7), which is reflected in the relatively small sensory differences. ME RB (+) wines were rated higher in “astringency/dry” mouthfeel compared to ME RB (−) wines, and this correlates with the higher concentration of tannins in ME RB (+) wines determined by protein precipitation assay compared to ME RB (−) wines (Figure 1).

It has been shown that lower pH and lower ethanol concentration increases “sourness” in dealcoholized white wine concentrate [25], which was the case of CS2 RB (+) wines in 2015. In addition, CS2 RB (+) wines were rated more “grippy”, a sub-term of astringency than RB (−) in 2015 (Supplemental Table S2) [50], which agrees with the higher concentration of tannin determined by the protein precipitation assay. The determination of tannin concentration in wines by protein precipitation has been shown to have the best correlation with astringency ratings in wines [51]. CS2 RB (−) wines were also rated higher than RB (+) wines regarding “sweet” taste in 2015 (Supplemental Table S2). This is partially explained because RB (−) wines have higher ethanol content than RB (+) wines. It has been found that alcohol enhanced the perception of sweetness in high alcohol wines when tasted after low alcohol wines [20]. However, in this study, the wines were presented in a randomized order during the DA evaluations. Therefore, other factors may have contributed to these findings, such as the higher content of monosaccharides in CS2 RB (−) wines. The levels of seven out of 32 monosaccharides (xylulose, xylonic acid, xylitol, ribonic acid, ketohexose, galactonic acid, and fructose) were significantly higher in RB (−) when compared with RB (+) wines for CS2 (2015 season) (Table 6). Monosaccharides have sweet detection thresholds of 10–50 mM, and the sweet taste of sugars increases the perception of wine body [9,13].

## 4. Material and Methods

### 4.1. Harvest and Winemaking

*Vitis vinifera* L. cv. Cabernet Sauvignon grapevines from two vineyards (CS1 and CS2), and *Vitis vinifera* L. cv. Merlot grapevines from one vineyard (ME), all in Napa Valley, were used for this investigation during the 2014 and 2015 seasons (Table 7). For all three vineyards, grapevines were trained to vertical shoot position (VSP), bilateral cordon, pruned to 2-bud spurs, thinned to two clusters per shoot (except weak shoots), and deficit irrigated was applied. Due to management practices that included diseased vine removal due to GRBV infection and spread, only fruit from CS2 site was available for winemaking in the 2015 season. From each vineyard, approximately 120 symptomatic “RB (+)” grapevines and 120 asymptomatic “RB (−)” were harvested in each season. Grapevines were monitored for several years prior to this study for the presence of GRBD symptoms to ensure healthy and disease status. Twenty percent of each RB (−) and RB (+) grapevines were tested by qPCR to ensure that the vines were either healthy or only infected with GRBV (data vines) and no other virus species were present. Grape samples were taken separately from data vines, and the larger set of 120 vines pool used for winemaking to ensure that they had similar chemical composition. A strict correlation between GRBV infection and appearance of symptoms has been demonstrated in *Vitis vinifera* grapevines [6].

For each site, approximately 400 kg of RB (+) and RB (−) grapes were manually harvested early in the morning when RB (−) grapes reached ~25 Brix and transported directly to the UC Davis Teaching and Research Winery (Davis, CA, USA). Grapes were destemmed and crushed using a Bucher Vaslin Delta E2 (Santa Rosa, CA, USA) destemmer/crusher directly into 200 L stainless steel research fermentors. Fifty mg/L of sulfur dioxide (SO_2_) was added and mixed by stirring in each tank prior to yeast inoculation. Fermentations were carried out in duplicate or triplicate depending on the quantity of grape available (CS1 and ME = duplicate; CS2 = triplicate).

*Saccharomyces cerevisiae* strain EC-1118 (Lallemand, Montreal, Canada) was used for inoculation according to the rehydration procedure described by the manufacturer. Prior to inoculation, diammonium phosphate (DAP) (Omnisal GmBH, Lutherstadt Wittenberg, Germany) and tartaric acid (American Tartaric Products, Windsor, CA, USA) were used to adjust yeast assimilable nitrogen (YAN) to 250 mg/L and titratable acidity (TA) to six g/L, respectively. Fermentation conditions were controlled by Integrated Fermentation Control System (IFCS) units (Cypress Semiconductor, San Jose, CA, USA) [34]. The temperature of fermentation was controlled at 25 °C, while cap management conditions were set to one tank volume pump-over twice a day. After eight days of maceration, the wines were dry (<2 g/L of sugar) and pressed using a basket press, then returned to the tanks, where they were inoculated with *Oenococcus oeni* to induce malolactic fermentation (MLF) (Chr. Hansen A/S, Hørsholm, Denmark). MLF was considered completed when malic acid levels were <0.2 g/L. Free SO_2_ was adjusted to 35 mg/L, and wines were racked to 49.2 L tanks and stored at 13 °C until bottling. The wines were bottled in Bordeaux style bottles with screw caps (Saranex/Transcendia, Franklin Park, IL, USA) and stored at 14 °C until analysis.

### 4.2. Basic Chemical Composition Determination

The chemical composition of the wines was analyzed at the time of bottling. Ethanol content was determined using an alcolyzer (Anton Parr, Ashland, VA, USA), while pH was determined with an Orion 5-star pH meter (Thermo Scientific, MA, USA). Titratable acidity, free and bound SO_2_ was measured with a Mettler-Toledo DL50 titrator (Mettler-Toledo Inc., Columbus, OH, USA). Residual sugar was measured using Thermo Scientific Gallery analyzer (Thermo Scientific, Waltham, MA, USA).

### 4.3. Total Phenolics, Total Tannin, and Total Anthocyanin Determination

The wines were analyzed by a modified tannin precipitation assay (Harbertson et al. 2015). Each wine was analyzed in triplicate using a Genesys10S UV-Vis Spectrophotometer (Thermo Fisher Scientific, Madison, WI, USA). This method determines total iron-reactive phenolics and total tannins at 510 nm expressed as catechin equivalents (CE), and total anthocyanins at 520 nm expressed as malvidin-3-glucoside (M3G) equivalents.

### 4.4. RP-HPLC Analysis of Phenolics

Frozen wine samples were thawed and centrifuged at 3200 times gravity (×*g*) for five minutes. The samples were analyzed by reversed phase-high performance liquid chromatography (RP-HPLC) using an Agilent 1260 Infinity equipped with a PLRP-S 100A 3um 150 × 4.6 mm column (Agilent Technologies, Santa Clara, CA, USA) at 35 °C, an autosampler with temperature control at 8 °C, and a diode array detector (DAD). In short, two mobile phases were used: mobile phase A (water containing 1.5% phosphoric acid *v*/*v*) and mobile phase B (80% acetonitrile and 20% mobile phase A). The gradient for separation used was described by Peng et al. [52]. Twenty µL of sample was injected with the mobile phase flow rate set at 1 mL/min. The eluted compounds were monitored and identified by spectral and retentions time comparisons to authentic standards and published literature at four different wavelengths: 280 nm (gallic acid, (+)-catechin, dimer B1, (−)-epicatechin, dimer B2, epicatechin gallate, and polymeric phenols), 320 nm (caftaric acid, caffeic acid, coutaric acid, *p*-coumaric acid), 360 nm (quercetin-3-galactoside, quercetin-3-glucuronide, quercetin-3-glucoside and quercetin-3-rhamnoside), and 520 nm (individual monomeric anthocyanins and polymeric pigments) [52]. External calibration curves were constructed using authentic standards; gallic acid, (+)-catechin, (−)-epicatechin, caffeic acid, quercetin, *p*-coumaric acid were purchased from Sigma-Aldrich (St. Louis, MO, USA), quercetin-rhamnoside and malvidin-3-*O*-glucoside chloride purchased from Extrasynthese (Genay, France). These compounds were quantified as themselves, whereas B1, epicatechin gallate, and polymeric phenols were quantified as (+)-catechin equivalents; caftaric acid as caffeic acid equivalents; coutaric acid as *p*-coumaric acid equivalents; quercetin-3-galactoside, quercetin-3-glucuronide, quercetin-3-glucoside as quercetin-3-rhamnoside equivalents; and anthocyanins and polymeric pigments as malvidin-3-*O*-glucoside equivalents. Data analysis was performed using Agilent^®^ CDS ChemStation software version D.04 (Agilent Technologies, Santa Clara, CA, USA).

### 4.5. Characterization of Proanthocyanidins

PA were isolated from wine using solid-phase extraction (SPE) as described by Oberholster et al. [9]. SPE was performed in triplicate using bed volumes of 10 mL of Toyopearl HW-40 size exclusion media. Wines were centrifuged at 3200× *g* for 15 min, and one mL of sample was loaded onto the SPE columns. The samples were washed using a 40 mL solution of ethanol:water (55:45) containing 0.05% trifluoroacetic acid (TFA). PA were eluted using 30 mL of a solution of acetone:water (60:40) containing 0.05% TFA. Collected PA extracts were concentrated under vacuum at 35 °C and reconstituted in 500 µL of methanol and stored at −20 °C until analysis.

The composition of isolated PA was determined according to the method described by Kennedy and Jones [53]. Phloroglucinolysis reaction products were analyzed by RP-HPLC using an Agilent Poroshell 120 SB-C18 (4.6 × 150 mm. 2.7 µm particle) HPLC column on an Agilent Infinity series 1260 HPLC system (Agilent Technologies, Inc., Deerfield, IL, USA) equipped with a DAD detector using a binary gradient with mobile phases of water containing 0.1% formic acid (mobile phase A) and acetonitrile containing 0.1% formic acid (mobile phase B). Twenty µL of sample was injected, and the eluted peaks were monitored at 280 nm. Gradient conditions were as follows: column temperature was kept at 35 °C, flow rate was 2 mL/min, 0–2.96 min, 3% B; 2.96–10.30 min, 3–16% B; 10.30–10.40 min, 16–20% B; 10.40–12.10 min, 20% B; 12.10–13.0 min, 20–80% B; 13.0–14.34 min, 80% B; 14.34–15.34 min, 80–3% B; 15.34–20.0 min, 3% B [54]. Data analysis was performed using Agilent^®^ CDS ChemStation software version D.04 (Agilent Technologies, Santa Clara, CA, USA) and quantified by external calibration with (+)-catechin using response factors relative to catechin [53]. For each wine sample, the mean degree of polymerization (mDP), PA concentration, % of galloylation, and % of gallo units was determined [9,53].

### 4.6. Metabolomics Analysis

Each fermentation replicate was analyzed in six replicates for untargeted metabolomics profiling performed by the Genomic Center at the University of California, Davis, using an automated Liner Exchange Gas Chromatography Time-of-Flight Mass Spectrometer (ALEX-GC-TOF-MS - Agilent Santa Clara, USA). Data acquisition and metabolite identification were performed as described in Fiehn et al. [55]. In summary, samples were derivatized by methoxyamine hydrochloride in pyridine and subsequently by *N*-methyl-*N*-trimethylsilyltrifluoroacetamide for trimethylsilylation of acidic protons. Compounds were identified by retention time and mass spectrum similarity compared to the Fiehn library containing 713 unique metabolites and 1197 unique spectra. A mix of internal retention index (RI) markers was prepared using fatty acid methyl esters (FAME) and used as internal standard (Fiehn et al. 2008).

Data were reported as peak height using the single ion as default after normalization to reduce the impact of between-series drifts of instrument sensitivity caused by machine maintenance, aging and tuning parameters. Peak heights were normalized by dividing each metabolite peak by the sum of all peak heights for all identified metabolites for each sample.

### 4.7. Descriptive Analysis

Descriptive analysis was performed in the J. Lohr Wine Sensory Room, University of California, Davis, approximately six months after the wines were bottled and within a month of chemical analysis in each season. Panelists were recruited by advertising within the campus of the University of California, Davis, and none of the panelists that participated were aware of the purpose of the study. The study was approved by the Institutional Review Board of the university (IRB ID 699890-1), and all panelists gave informed oral consent. Twelve volunteer panelists participated in the study in 2014 (seven males and five females) and 10 panelists in 2015 (six males and four females). During seven training sessions of one hour each, panelists generated sensory attributes by consensus, as well as the related reference standards (Table 8 and Table 9). After the training session, the wines were rated in triplicate during six evaluation sessions with seven wines. Panelists rated aroma, taste, and mouthfeel attributes intensities using a 15-cm unstructured line scale anchored with the terms “none” and “very intense”, except for “viscous”, in which the anchors were “watery” and “very viscous”. Prior to each evaluation session, the panelists were tested to recognize all smell reference standards blind to ensure the memorization of all attributes. The presentation order of the wines was randomized according to a Latin Square Design. Wines were served in black International Standards Organization (ISO) wine tasting glasses covered with plastic Petri dishes. Thirty milliliters of the wine was poured no more than 15 min before they were tasted. Glasses were labeled with three-digit random numbers. Panelists were instructed to expectorate the samples. To reduce carryover, a one-minute break was requested before evaluating the next wine, where water and unsalted crackers were served to cleanse the palate. Data acquisition for all sensory experiments was carried out using FIZZ software (FIZZ network, version 2.47 B, Biosystems, Courtenon, France).

### 4.8. Statistical Analysis

All chemical, phenolic and metabolomic data were analyzed using univariate analyses of variance (ANOVA) measuring the effect of the treatments. The differences between treatment means were determined by Fisher’s least significant differences (LSD). Sensory data were tested for significance by multivariate analysis of variance (MANOVA) for the overall treatment effect. Univariate analyses of variance (ANOVA) was performed for those attributes that showed differences in the overall treatment effect. Principal component analysis (PCA) was performed to compare graphically the relations between RB (−) and RB (+) wines regarding sensory attributes and chemical composition. Statistical significance was set at 5%. All statistical analyses were performed using XLSTAT (Microsoft Office Professional Plus 2010, version 14.0.7194.5000, Redmond, WA, USA).

## 5. Conclusions

This study aimed to quantify the impact of GRBD on wine composition and sensory characteristics for different sites across two seasons. Data obtained in this study suggest that there is a link between the extent of GRBD impact on grape ripening (mainly observed on sugar accumulation and as a consequence in wine ethanol content) and wine metabolite concentrations derived from grape berry and fermentation products. The degree of GRBD impact on grape and wine composition was variable and depended on the site and season, although the latter could only be investigated for one site. Consistently, RB (+) wines contained less ethanol when compared to RB (−) wines due to lower sugar content in the corresponding grapes.

Regarding phenolic composition, differences between RB (−) and RB (+) wines were variable and depended on the site and season. In general, GRBD impacted RB (+) wines by reducing anthocyanin content, in some cases, and increasing flavonol and PA concentration when compared to RB (−) wines. Wine sensory attributes, such as aroma, taste, and mouthfeel, were altered due to GRBD with a large impact due to ethanol differences. This study is a first screening of the impact of GRBD on wine composition and subsequent sensory attributes and aimed to assess the differences between RB (−) and RB (+) wines from each site. Additional studies are necessary to identify the specific influence of site characteristics, such as irrigation regime, vine age, nutrient management, and rootstock characteristics, on the impact of GRBD on grapes and the resulting wines. In addition, more research is needed to develop potential mitigation strategies in both the vineyard and the winery.

## Figures and Tables

**Table 1:** Must composition at harvest of RB (−) and RB (+) wines in 2014 and 2015.

Year	Grape/RB Status	Harvest	TSS (Brix)	pH	TA (g/L)	YAN (mg/L)	Malic Acid (mg/L)
2014	CS1 RB (−)	19 September 2014	24.20 ± 0.28 a	3.55 ± 0.01 a	3.59 ± 0.02 b	126.37 ± 5.28 a	1088.50 ± 51.62 a
	CS1 RB (+)	19 September 2014	21.85 ± 0.07 b	3.51 ± 0.02 b	3.83 ± 0.01 a	124.05 ± 0.59 a	1111.50 ± 38.89 a
	CS2 RB (−)	07 October 2014	26.27 ± 0.15 a	3.59 ± 0.01 a	4.84 ± 0.09 a	187.37 ± 5.92 a	1502.00 ± 30.81 a
	CS2 RB (+)	07 October 2014	25.20 ± 0.10 b	3.55 ± 0.01 b	4.87 ± 0.11 a	170.71 ± 4.79 b	1430.67 ± 37.45 a
	ME RB (−)	26 September 2014	24.90 ± 0.28 a	3.53 ± 0.01 a	4.15 ± 0.16 b	68.67 ± 1.53 a	895.50 ± 27.58 b
	ME RB (+)	26 September 2014	23.45 ± 0.10 b	3.47 ± 0.01 b	4.74 ± 0.02 a	37.06 ± 0.59 b	1021.50 ± 9.19 a
2015	CS2 RB (−)	21 September 2015	26.00 ± 0.26 a	3.73 ± 0.07 a	4.32 ± 0.08 a	197.29 ± 3.94 a	2594 ± 51.88 b
	CS2 RB (+)	21 September 2015	22.40 ± 0.22 b	3.71 ± 0.06 a	4.45 ± 0.07 a	168.42 ± 3.36 b	2303 ± 46.06 a

Table shows the mean ± standard deviation of fermentation replicates (n = 3, *p* < 0.05). Statistical differences are expressed as letters and indicate significant differences in the least significant differences (LSD) test. Means within a column followed by the same letter are not significantly different within each site and year. a CS1 = Cabernet Sauvignon—Site 1. b CS2 = Cabernet Sauvignon—Site 2. ME = Merlot. RB (−) = Wine made with grapes from healthy grapevines. RB (+) = Wines made with grapes from grapevines symptomatic for grapevine red blotch disease (GRBD). TA = Titratable acidity expressed in g/L of tartaric acid. YAN = Yeast assimilable nitrogen. TSS = Total soluble solids.

**Table 2:** Basic chemical composition of RB (−) and RB (+) wine in 2014 and 2015.

Year	Wine/RB Status	Ethanol (%)	pH	TA (g/L)	Residual Sugar (g/L)
2014	CS1 RB (−)	14.64 ± 0.33 a	3.22 ± 0.01 a	7.35 ± 0.04 a	0.08 ± 0.00 a
	CS1 RB (+)	12.97 ± 0.08 b	3.19 ± 0.05 a	7.07 ± 0.41 a	0.10 ± 0.03 a
	CS2 RB (−)	15.79 ± 0.10 a	3.90 ± 0.01 a	4.85 ± 0.03 b	0.33 ± 0.02 a
	CS2 RB (+)	14.89 ± 0.03 b	3.73 ± 0.01 b	5.53 ± 0.01 a	0.24 ± 0.02 b
	ME RB (−)	15.26 ± 0.05 a	3.66 ± 0.01 b	5.22 ± 0.08 a	0.18 ± 0.01 a
	ME RB (+)	14.08 ± 0.11 b	3.73 ± 0.01 a	5.32 ± 0.01 a	0.13 ± 0.00 b
2015	CS2 RB (−)	15.13 ± 0.08 a	3.82 ± 0.02 a	5.56 ± 0.05 b	0.20 ± 0.02 b
	CS2 RB (+)	12.89 ± 0.11 b	3.62 ± 0.01 b	6.00 ± 0.01 a	0.40 ± 0.02 a

Table shows the mean ± standard deviation of fermentation replicates (n = 3 for CS2 and n = 2 for CS1 and ME, *p* < 0.05). Statistical differences are expressed as letters and indicate significant differences in the LSD test. Means within a column followed by the same letter are not significantly different within each site and year. a CS1 = Cabernet Sauvignon—Site 1. b CS2 = Cabernet Sauvignon—Site 2. ME = Merlot. RB (−) = Wine made with grapes from healthy grapevines. RB (+) = Wines made with grapes from grapevines symptomatic for GRBD. TA = Titratable acidity.

**Table 3:** Phenolic profiles of RB (−) and RB (+) wines over two seasons.

Compound	2014							2015
	CS1 RB (–)	CS1 RB (+)	CS2 RB (−)	CS2 RB (+)	ME RB (−)	ME RB (+)	CS2 RB (−)	CS2 RB (+)
Gallic acid	11.53 ± 0.22 a	9.37 ± 0.27 a	13.39 ± 0.18 a	13.21 ± 0.12 a	19.09 ± 0.93 a	19.72 ± 0.94 a	14.51 ± 0.23 b	17.41 ± 0.52 a
(+)-Catechin	9.92 ± 0.00 a	10.88 ± 1.06 a	16.59 ± 0.48 a	14.71 ± 0.86 b	29.70 ± 0.17 b	35.50 ± 0.04 a	21.74 ± 0.61 a	24.06 ± 2.4 a
(–)-Epicatechin	2.41 ± 0.34 a	2.32 ± 0.33 a	7.36 ± 0.86 a	4.39 ± 0.89 a	12.75 ± 0.14 a	12.81 ± 0.40 a	0.17 ± 0.02 a	0.24 ± 0.04 a
**Total flavan-3-ols**	**41.14 ± 0.43 a**	**42.87 ± 1.20 a**	**66.76 ± 1.93 a**	**59.44 ± 3.04 b**	**81.95 ± 0.19 b**	**101.72 ± 0.28 a**	**40.58 ± 1.75 a**	**44.59 ± 3.59 a**
Caftaric acid	2.65 ± 0.19 a	3.81 ± 0.58 a	-	-	2.57 ± 0.17 a	1.68 ± 0.21 b	-	-
Caffeic acid	19.07 ± 5.94 a	23.47 ± 0.29 a	17.55 ± 0.43 a	14.85 ± 0.81 b	30.29 ± 0.77 a	32.71 ± 0.87 a	6.28 ± 0.60 a	4.15 ± 0.76 b
Coutaric acid	1.35 ± 0.06 a	2.02 ± 0.20 a	-	-	1.05 ± 0.02 a	0.79 ± 0.01 b	-	-
*p*-Coumaric acid	6.58 ± 1.94 a	8.54 ± 0.09 a	8.78 ± 0.21 a	7.56 ± 0.71 b	8.37 ± 0.32 a	8.76 ± 0.33 a	3.20 ± 0.42 a	2.24 ± 0.53 a
**Total hydroxycinnamic acid**	**29.67 ± 8.14 a**	**37.86 ± 0.40 a**	**26.34 ± 0.38 a**	**22.41 ± 1.48 b**	**42.29 ± 0.64 a**	**43.95 ± 0.98 a**	**9.48 ± 1.01 a**	**6.40 ± 1.28 b**
Quer-galactoside	2.66 ± 0.01 a	2.97 ± 0.26 a	1.85 ± 0.07 b	2.44 ± 0.09 a	5.28 ± 0.26 a	5.51 ± 0.16 a	1.61 ± 0.23 a	1.35 ± 0.08 a
Quer-3-glucoside	11.34 ± 0.48 a	11.80 ± 1.61 a	15.52 ± 0.71 b	18.64 ± 0.54 a	32.83 ± 1.47 b	37.02 ± 1.06 a	3.44 ± 0.76 a	3.37 ± 0.2 a
Quer-glucuronide	22.99 ± 0.38 a	24.17 ± 0.33 a	18.36 ± 0.83 a	20.52 ± 1.92 a	26.33 ± 2.78 a	27.95 ± 1.08 a	12.71 ± 1.07 a	12.20 ± 0.6 a
Quer-rhamnoside	14.11 ± 0.02 a	13.03 ± 0.29 b	16.90 ± 0.43 a	16.12 ± 0.06 a	10.75 ± 0.72 a	10.19 ± 0.30 a	8.90 ± 0.24 a	7.03 ± 0.2 b
Quercetin	8.64 ± 0.23 a	8.04 ± 0.62 a	6.01 ± 2.00 b	8.25 ± 0.47 a	11.43 ± 0.93 a	11.30 ± 0.51 a	3.29 ± 0.71 a	3.94 ± 0.22 a
**Total flavonols**	**62.86 ± 0.35 a**	**62.08 ± 2.46 a**	**61.61 ± 0.30 b**	**67.63 ± 0.36 a**	**86.87 ± 4.00 a**	**91.98 ± 3.13 a**	**29.95 ± 2.05 a**	**27.89 ± 1.15 a**
**Polymeric phenols**	**515.40 ± 46.53 a**	**327.32 ± 0.07 b**	**459.92 ± 100.10 a**	**374.68 ± 9.77 b**	**395.97 ± 10.40 a**	**449.41 ± 26.84 a**	**149.96 ± 7.66 a**	**146.32 ± 4.91 a**
Delph-3-gluc	7.18 ± 0.02 a	6.92 ± 0.54 a	7.31 ± 0.27 a	6.36 ± 0.25 b	28.76 ± 0.38 a	24.19 ± 0.62 b	4.88 ± 0.07 a	3.4 ± 0.23 b
Cya-3-gluc	-	-	-	-	5.45 ± 0.04 a	4.25 ± 0.20 b	0.22 ± 0.02 a	0.16 ± 0.01 b
Pet-3-gluc	7.57 ± 0.12 a	7.69 ± 0.01 a	10.52 ± 0.38 a	9.09 ± 0.37 b	27.17 ± 0.51 a	24.60 ± 0.53 b	7.98 ± 0.13 a	5.53 ± 0.42 b
Peo-3-gluc	5.27 ± 0.01 a	5.68 ± 0.06 a	4.97 ± 0.21 a	4.73±0.08 a	20.92 ± 0.12 a	15.67 ± 0.36 b	3.57 ± 0.05 a	3.05 ± 0.21 b
Malv-3-gluc	84.51 ± 6.06 a	107.36 ± 11.75 a	167.85 ± 5.98 a	147.69±4.83 b	100.85 ± 0.27 b	112.88 ± 1.49 a	153.13 ± 2.97 a	127.22 ± 6.36 b
Delph-3-glu-ac	1.76 ± 0.17 a	1.94 ± 0.04 a	4.10 ± 0.25 a	2.98 ± 0.27 b	8.34 ± 0.18 a	6.90 ± 0.10 b	2.14 ± 0.21 a	1.39 ± 0.2 b
Pet-3-glu-ac	1.86 ± 0.05 a	1.88 ± 0.15 a	3.00 ± 0.12 a	2.44 ± 0.18 b	6.28 ± 0.10 a	5.50 ± 0.02 b	2.91 ± 0.14 a	2.04 ± 0.15 b
Peo-3-glu-ac	1.64 ± 0.05 a	2.18 ± 0.26 a	2.38 ± 0.05 a	2.09 ± 0.10 b	5.84 ± 0.01 a	5.02 ± 0.12 b	1.41 ± 0.05 a	1.33 ± 0.08 a
Malv-3-glu-ac	27.60 ± 2.27 a	41.67 ± 7.06 a	63.26 ± 1.27 a	56.02 ± 3.37 b	25.48 ± 0.61 b	31.25 ± 0.09 a	55.65 ± 1.47 a	54.38 ± 3.73 a
Peo-3-glu-cou	0.60 ± 0.06 b	0.94 ± 0.07 a	0.45 ± 0.05 a	0.42 ± 0.02 a	3.40 ± 0.03 a	2.74 ± 0.00 b	0.48 ± 0.04 a	0.42 ± 0.05 a
Malv-3-glu-cou	8.37 ± 0.83 b	13.17 ± 1.78 a	11.54 ± 0.28 a	11.62 ± 0.68 a	11.85 ± 0.05 b	16.25 ± 0.37 a	8.92 ± 0.46 a	9.64 ± 0.97 a
**Total anthocyanin**	**146.40 ± 9.59 a**	**189.48 ± 20.46 a**	**275.42 ± 8.31 a**	**243.50 ± 10.06 b**	**245.41 ± 1.24 a**	**250.13 ± 3.18 a**	**242.71 ± 1.63 a**	**210.06 ± 12.34 b**
**Polymeric pigments**	**39.72 ± 3.37 a**	**24.35 ± 2.23 b**	**23.183 ± 1.70 a**	**19.338 ± 0.78 b**	**19.89 ± 1.11 a**	**18.94 ± 1.13 a**	**12.76 ± 0.62 a**	**10.09 ± 0.2 b**

Table shows the mean ± standard deviation of fermentation replicates (n = 3 for CS2 and n = 2 for CS1 and ME, *p* < 0.05). Cabernet Sauvignon Site 1 (CS1), Cabernet Sauvignon Site 2 (CS2) and Merlot (ME) concentration of 2014 wines (n = 3, *p* < 0.05). Statistical differences are expressed as different letters within each site and year and indicate significant differences in the LSD test. a CS1 = Cabernet Sauvignon—Site 1. b CS2 = Cabernet Sauvignon—Site 2. ME = Merlot. RB (−) = Wine made with grapes from healthy grapevines. RB (+) = Wines made with grapes from grapevines symptomatic for GRBD.

**Table 4:** Proanthocyanidin (PA) composition of RB (−) and RB (+) wines in 2014 and 2015 seasons.

Variable	2014						2015	
	CS1 RB (−)	CS1 RB (+)	CS 2 RB (−)	CS2 RB (+)	ME RB (−)	ME RB (+)	CS2 RB (−)	CS2 RB (+)
EGC-P	1.90 ± 0.02 a	2.65 ± 0.27 a	2.43 ± 0.26 b	3.07 ± 0.16 a	3.97 ± 0.30 b	4.80 ± 0.34 a	1.19 ± 0.33 a	1.76 ± 0.28 a
C-P	0.09 ± 0.00 a	0.11 ± 0.00 a	0.13 ± 0.01 b	0.17 ± 0.01 a	0.35 ± 0.02 b	0.55 ± 0.03 a	0.23 ± 0.02 b	0.31 ± 0.03 a
EC-P	2.18 ± 0.08 a	2.76 ± 0.24 a	3.06 ± 0.28 b	3.83 ± 0.35 a	6.17 ± 0.51 b	9.35 ± 0.67 a	2.90 ± 0.29 b	4.08 ± 0.24 a
ECG-P	0.08 ± 0.00 a	0.10 ± 0.00 a	0.10 ± 0.01 a	0.12 ± 0.01 a	0.27 ± 0.03 a	0.36 ± 0.03 a	0.17 ± 0.02 b	0.27 ± 0.02 a
EGC	-	-	-	-	-	-	-	-
C	0.24 ± 0.01 a	0.31 ± 0.02 a	0.33 ± 0.03 b	0.39 ± 0.03 a	0.77 ± 0.05 b	1.16 ± 0.06 a	0.36 ± 0.02 b	0.47 ± 0.05 a
EC	0.05 ± 0.00 b	0.07 ± 0.00 a	0.07 ± 0.00 b	0.09 ± 0.00 a	0.22 ± 0.02 b	0.28 ± 0.01 a	0.03 ± 0.01 a	0.02 ± 0.00 a
ECG	0.01 ± 0.00 a	0.01 ± 0.00 a	0.00 ± 0.00 a	0.00 ± 0.00 a	0.01 ± 0.00 a	0.01 ± 0.00 a	0.00 ± 0.00 b	0.01 ± 0.00 a
Terminal units	0.31 ± 0.01 a	0.40 ± 0.02 a	0.42 ± 0.04 b	0.50 ± 0.04 a	1.02 ± 0.08 b	1.47 ± 0.08 a	0.39 ± 0.03 b	0.5 ± 0.05 a
Extension units	4.26 ± 0.11 a	5.63 ± 0.52 a	5.74 ± 0.51 b	7.19 ± 0.51 a	10.78 ± 0.80 b	15.06 ± 1.08 a	4.48 ± 0.65 b	6.42 ± 0.15 a
Term + Ext	4.58 ± 0.11 a	6.03 ± 0.54 a	6.17 ± 0.54 b	7.69 ± 0.54 a	11.80 ± 0.95 b	16.53 ± 1.14 a	4.87 ± 0.68 b	6.92 ± 0.18 a
mDP	14.61 ± 0.62 a	15.03 ± 0.72 a	14.51 ± 1.05 b	15.41 ± 0.53 a	11.50 ± 0.26 a	11.24 ± 0.47 a	12.53 ± 0.87 a	13.88 ± 1.14 a
PA (mg/L)	204.82 ± 5.32 b	269.73 ± 24.35 a	275.08 ± 24.14 b	343.15 ± 24.38 a	526.52 ± 42.73 b	734.82 ± 51.20 a	434.8 ± 61.23 b	619.1 ± 15.79 a
Galloylation%^o^	2.23 ± 0.15 a	1.91 ± 0.185 a	1.86 ± 0.09 a	1.75 ± 0.15 a	2.46 ± 0.09 a	2.27 ± 0.13 b	3.60 ± 0.06 b	3.98 ± 0.22 a
% Gallo units	41.59 ± 0.58 b	43.88 ± 0.84 a	39.38 ± 2.36 a	39.93 ± 1.70 a	33.66 ± 0.29 a	29.03 ± 0.36 b	23.97 ± 3.48 a	25.38 ± 4.1 a
Average MW	4357.7 ± 187.9 a	4479.3 ± 216.1 a	4315.0 ± 316.3 b	4581.3 ± 161.4 a	3418.8 ± 79.2 a	3331.2 ± 142.3 a	3730.3 ± 263.3 a	4139.7 ± 345.2 a

Table shows the mean ± standard deviation of fermentation replicates (n = 3 for CS2 and n = 2 for CS1 and ME, *p* < 0.05). Statistical differences are expressed as different letters and indicate significant differences in the LSD test within each site and year. CS1 = Cabernet Sauvignon—Site 1. CS2 = Cabernet Sauvignon—Site 2. ME = Merlot. RB (−) = Wine made with grapes from healthy grapevines. RB (+) = Wines made with grapes from grapevines symptomatic for GRBD. mDP = Mean degree of polymerization of tannin, calculated by the sum of all subunits (flavan-3-ol monomer and phloroglucinol adduct, in moles), divided by the sum of all flavan-3-ol monomers (in moles). MW = Molecular weight of tannin. P = phloroglucinol adduct of extension subunit (in moles). EGC = Epigallocatechin (in moles). C = Catechin (in moles). EC = Epicatechin (in moles). ECG = Epicatechin gallate tannin subunits (in moles). PA = Proanthocyanidin. Galloylation% = Percentage galloylated units (ECG and ECG-P) of the total. % Gallo units = Percentage of gallo units (EGC-P and EGC) of the total.

**Table 5:** Primary and secondary metabolites significantly impacted by GRBD as determined by untargeted metabolomics profiling for 2014 wines.

Compound	ME	CS 1	CS 2	Compounds	ME	CS 1	CS 2
***Amino Acid***				***Carboxylic Acid***			
xanthine				vanillic acid			
valine				threonic acid			
uracil				tartaric acid			
tyrosine				sulfuric acid			
tryptophan				succinic acid			
trans-4-hydroxyproline				shikimic acid			
thymine				pyruvic acid			
threonine				pyrrole-2-carboxylic acid			
spermidine				pipecolinic acid			
serine				mucic acid			
proline				malonic acid			
oxoproline				malic acid			
nicotianamine				lactic acid			
methionine				isohexonic acid			
lysine				isocitric acid			
isoleucine				hexuronic acid			
homoserine				glycolic acid			
histidine				gluconic acid lactone			
guanidinosuccinate				fumaric acid			
glycine				dehydroascorbic acid			
glutaric acid				citric acid			
glutamine				citramalic acid			
gluconic acid				cis-caffeic acid			
beta-alanine				benzoic acid			
aspartic acid				aconitic acid			
alpha-ketoglutarate				4-hydroxycinnamic acid			
alanine-alanine				4-hydroxybutyric acid			
alanine				3-phenyllactic acid			
4-aminobutyric acid				3-hydroxypropionic acid			
cysteine				3-hydroxy-3-methylglutaric acid			
ornithine				3,4-dihydroxycinnamic acid			
glutamic acid				3,4-dihydroxybenzoic acid			
asparagine				2-isopropylmalic acid			
				2-hydroxyglutaric acid			
				2-deoxytetronic acid			
				glyceric acid			
***Monosaccharide***				***Polyol***			
ribose				conduritol-beta-epoxide			
tagatose				threitol			
xylulose				sorbitol			
xylose				ribitol			
xylonic acid				quinic acid			
xylitol				pentitol			
ribonic acid				lyxitol			
n-acetylputrescine				glycerol-alpha-phosphate			
myo-inositol				galactinol			
levoglucosan				erythritol			
ketohexose				deoxypentitol			
hexitol				6-deoxyglucitol			
glycerol-3-galactoside				2-deoxyerythritol			
glucose-1-phosphate				mannitol			
glucose				glycerol			
glucoheptulose				butane-2,3-diol			
galacturonic acid				2-deoxypentitol			
galactonic acid				1,2-anhydro-myo-inositol			
fucose				***Flavonoid***			
fructose				tyrosol			
dihydroxyacetone				gallocatechin			
digitoxose				epicatechin			
6-deoxyglucose				catechin			
galactose				***Lipid***			
erythrose				capric acid			
***Oligosaccharide***				stearic acid			
sophorose				pelargonic acid			
digalacturonic acid				palmitic acid			
trehalose				ethylsuccinate nist			
sucrose				phytol			
isomaltose				oleic acid			
cellobiose				octanol nist			
beta-gentiobiose				octadecanol			
maltotriose				lauric acid			
leucrose				heptadecanoic acid			
	Significantly higher for RB (+) grapes when compared to RB (−) within the site.						
	Significantly higher for RB (−) grapes when compared to RB (+) within the site.						
	No significant differences between RB (+) and RB (−) within the site. (n=18, *p* < 0.05).						

**Table 6:** Primary and secondary metabolites impacted by GRBD as determined by untargeted metabolomics for CS2 2015 wines.

Compounds	CS 2	Compounds	CS 2	Compounds	CS 2	Compounds	CS 2
Amino Acids		Carboxylic Acids		Monosaccharide		Flavonoids	
xanthine		threonic acid		ribose		tyrosol	
valine		tartaric acid		xylulose		ferulic acid	
uracil		succinic acid		xylose		epigallocatechin	
tyrosine		shikimic acid		xylonic acid isomer		epicatechin	
tryptophan		pyruvic acid		xylitol		catechin	
trans-4-hydroxyproline		pyrrole-2-carboxylic acid		ribonic acid			
thymine		pipecolinic acid		N-acetylmannosamine		Lipids	
threonine		p-hydroxylphenyllactic acid		n-acetyl-d-hexosamine		capric acid	
spermidine		pentonic acid		myo-inositol		stearic acid	
serine		mucic acid		mannose		pelargonic acid	
sarcosine		mannonic acid NIST		lyxose		palmitic acid	
saccharopine		malonic acid		levoglucosan		myristic acid	
proline		malic acid		ketohexose		hexadecylglycerol	
phenylalanine		lactic acid		isoribose		ethylsuccinate	
pantothenic acid		keto-hexonic acid		hexose		Polyol	
oxoproline		isohexonic acid		hexitol		threitol	
nicotianamine		isocitric acid		glycerol-3-galactoside		sorbitol	
N-acetylputrescine		hexuronic acid		glucose-1-phosphate		ribitol	
N-acetyl-D-mannosamine		glycolic acid		glucose		quinic acid	
methionine		glutaric acid		gluconic acid		propane-1,3-diol NIST	
lysine		gluconic acid lactone		glucoheptulose		pentitol	
isoleucine		fumaric acid		galacturonic acid		maltitol	
hypoxanthine		dehydroascorbic acid		galactonic acid		lyxitol	
homoserine		citric acid		fucose		isothreitol	
homocystine		citramalic acid		fructose		hexadecane	
histidine		cis-caffeic acid		dihydroxyacetone		glycerol-alpha-phosphate	
guanidinosuccinate		benzoic acid		digitoxose		galactinol	
glycine		aconitic acid		arabinose		erythritol	
glutaric acid		4-hydroxycinnamic acid		6-deoxyglucose		deoxypentitol	
glutamine		4-hydroxybutyric acid		3,6-anhydro-d-hexose		arabitol	
glutamic acid		3-phenyllactic acid		3,6-anhydro-D-glucose		6-deoxyhexitol NIST	
beta-alanine		3-hydroxypropionic acid				6-deoxyglucitol	
aspartic acid		3-hydroxy-3-methylglutaric acid		Oligosaccharide		2-deoxyerythritol	
asparagine		3,4-dihydroxycinnamic acid		digalacturonic acid		1,2-anhydro-myo-inositol	
alpha-ketoglutarate		3,4-dihydroxybenzoic acid		trisaccharide			
alanine-alanine		2-isopropylmalic acid		trehalose			
alanine		2-hydroxyhexanoic acid		sucrose			
adenine		2-hydroxyglutaric acid		isomaltose			
4-aminobutyric acid		2,3-dihydroxybutanoic acid		melezitose			
cysteine		glyceric acid		cellobiose			
	Significantly higher for RB (+) grapes when compared to RB (−) within site.						
	Significantly higher for RB (−) grapes when compared to RB (+) within site.						
	No significant differences between RB (+) and RB (−) within site. (n=18, *p* < 0.05).						

**Table 7:** American Viticultural Area (AVA), county, season, cultivar, rootstocks, and age of the vineyards used for the study.

AVA	County	Season	Cultivar	Name	Rootstock	Planted
Oakville	Napa	2014	Cabernet Sauvignon 1	CS1	110R	2007
Rutherford	Napa	2014/2015	Cabernet Sauvignon 2	CS2	039-16	2010
Oakville	Napa	2014	Merlot	ME	* Riparia gloire*	1999

**Table 8:** Attributes used in the descriptive analysis (DA) and the corresponding reference standards in 2014.

Attribute	Reference
**Aroma**	
Red fruits	1 chopped cherry + 2 g chopped raspberry + 4 g strawberry (fresh frozen) + 5 g cranberry sauce (Ocean Spray, Middleborough, MA USA)
Dark fruits	2 g red plum jam (Smucker’s, Orrville, OH, USA) + 1 frozen blueberry (BestYet, Bethpage, NY, USA) + 1,5 mL black cherry juice (Lakewood, Miami, FL, USA)
Dried fruits	1/4 prune (Newman´s Own) + 1 dried cranberry (Ocean Spray Craisins) + 1 dried cherry (Mariani, Vacaville, CA, USA) + 1 raisin
Oxidized apple	2.5 mL apple juice (Minute Maid, Sugar Land, TX, USA, 100% apple juice) + 1 mL Tio Pepe Jerez Sherry (Palomino Fino)
Jammy	2 g dark cherry jam (d´arbo Marasque sour cherry fruit spread) + 2 g red plum jam (Smucker´s)
Cooked vegetables/green bell pepper	2.5 g fresh green bell pepper + 5 mL green bean brine (Del Monte cut green beans)
Leafy/tobacco	0.1 g tobacco (Malborough, South Hams, UK) + 0.2 g chopped leaf (Maple, BC, Canada), extract for 30 min
Earthy/leathery/mineral	1 hint of earth, 5 min + 0.5 cm leather shoe lace + 1 large gravel
Cedar	5 drops of ethanolic cedar extract
Oaky	0.5 cm^2^ oak chip (American oak, medium toast), split, extract for 30 min
Alcohol	1.5 mL vodka (Seagram´s extra smooth vodka)
Solvent/sulfur	100 µL SO2 Solution (15%) + 1 drop of nail polish remover (Salon Plus)
Baking spices	1 hint of pumpkin pie spice (Mc Cormick, Baltimore, MD, USA) + 0.5 cm vanilla bean (organics)
Black pepper	1 hint of freshly ground plack pepper (Mc Cormick)
Cacao/chocolate	0.05 g chocolate shavings (Trader Joe´s Mini, Pronto, LA, USA (70%, cacao dark chocolate bars))
Floral	2 petals of a dried rose bud (Co-op 5503) + 1 tip of fresh lavender, extract for 30 min
**Taste and Mouthfeel**	
Sweet	7 g/L sucrose C&H
Sour	2 g/L tartaric acid (Sigma Aldrich, St. Louis, MO, USA)
Bitter	0.8 g/L caffeine
Salty	3 g/L salt (Kosher salt, Morton, Kassel, Germany)
Coating	2 g/L carboxymethyl cellulose (Sigma Aldrich)
Viscous	1.25 g CMC/250 mL of water
Astringent/dry	1 g/l proanthocyanidic tannins extracted from grapes (Biotan, Laffort, Bordeaux, France) + 0.4 g/L alum (Mc Cormick)
Grippy	Tannic acid (1g/L)
Hot/alcohol	150 mL/L Vodka (Seagram’s extra smooth Vodka)

All aroma references were prepared in 10 mL base wine (Franzia Burgundy, Burgundy, France). All taste and mouthfeel references were prepared in filtered water (Arrowhead, San Bernardino Mountains, CA, USA).

**Table 9:** Attributes used in the descriptive analysis (DA) and the corresponding reference standards in 2015.

Attribute	Reference
**Aroma**	
Stone fruit	1/4 nectarine (fresh frozen) and 1/2 peach (365 sliced peach) in 20 mL wine
Blackberry	3 frozen blackberries and 2 g Bonne Maman blackberry preserve in 20 mL wine
Prune/dried fruit/honey	1/4 prune (Newmans own), 4 mL prune juice (Newmans own), 1 raisin (Sunmaid), 1 dried cranberry (Trader Joes), 2 dried blueberries (Kirkland), 1/2 dried white fig (Whole Foods), 1 g honey in 20 mL wine
Floral	2 rose petals, 1 lavender tip, steeped for 30 min in 20 mL wine
Green bell pepper	2.5 g diced green bell pepper steeped for 30 min in 20 mL wine
Citrus	1.5 g lemon in wine, 2 g orange in wine in 20 mL wine
Cherry/cranberry	3 g dried cherry, 1/2 cherry ice cube, 3 g d’arbo cherry jam, 5 g frozen cherries in 20 mL wine
Chocolate/cocoa	0.2 g Scharffen Berger 82% chocolate, 0.2 g Ghirardelli natural unsweetened cocoa in 20 mL wine
Pepper	2 hints ground black pepper in wine in 20 mL wine
Tomato/thyme	10 g sign chopped tomato (Signature Kitchen), 0.03 g dried Mc Cormick thyme in 20 mL wine
Soy	3 drops shoyu (San-j Organic) in 20 mL wine
Oak	0.1g french oak large chips medium toast in 20 mL wine
Alcohol	6 mL vodka (Seagrams) in 20 mL wine
Acetone	20 drops nail polish remover (Pretty Nails) in 20 mL wine
Elderberry	3 drops elderberry syrup (Davis Food Co-op Sambucus) in 20 mL wine
Eucalyptus	2 drops diluted eucalyptus oil (1 drop eucalyptus oil in 50mL water) in 20 mL wine
**Taste and Mouthfeel**	
Sour	2 g/L tartaric acid in water
Sweet	7 g/L sugar in water
Effervescent	Crystal geyser sparkling water
Viscous	2 g/L carboxymethylcellulose in water
Hot	500 mL/L Seagrams vodka in water
Umami	8 g/L MSG in water
Bitter	0.8 g/L caffeine in water
Coarseness	Texture of the wine in the mouth from fine grade sand paper to coarse
Grippy	Danon plain non-fat yogurt, experienced after expectoration
Drying	0.6 g/L alum in water

All aroma references using wine were prepared with Franzia Burgundy as base wine. All taste and mouthfeel references were prepared in filtered water (Arrowhead).

## Supplementary Materials

The following are available online. Figure S1: Cabernet Sauvignon Site 1 (CS1), Cabernet Sauvignon Site 2 (CS2), and Merlot (ME) alcoholic fermentation profile of wines from 2014 season, Figure S2: Cabernet Sauvignon Site 2 (CS2) total phenolics, total anthocyanin, and total tannins concentration of 2015 wines, Table S1: Growing degree days (GDD) and precipitation (mm) accumulated, average air temperature maximum and minimum at Oakville Weather Station from April 1st to October 31st on the 2014 and 2015 seasons, Table S2: Descriptive analysis scores of RB (−) and RB (+) wines from 2014 and 2015.
